# Low-energy Pulsed Electromagnetic Field Therapy Reduces Pain in Fibromyalgia: A Randomized Single-blind Controlled Pilot Study

**DOI:** 10.2478/rir-2022-0013

**Published:** 2022-07-06

**Authors:** Massimo Giovale, Lucia Novelli, Luca Persico, Francesca Motta, Stefano Rampoldi, Rossana Galli, Patrizia Monteforte, Marica Doveri, Gerolamo Bianchi, Carlo Selmi, Luigi Carlo Bottaro

**Affiliations:** 1 Division of Rheumatology, ASL3 Genovese, Genoa, Italy; 2 Rheumatology and Clinical Immunology; IRCCS Humanitas Research Hospital, Rozzano, Milan, Italy; 3 Department of Economics, University of Genova, Genoa, Italy; 4 Department of Biomedical Sciences, Humanitas University, Milan, Italy; 5 THS Therapeutic Solutions srl, Milan, Italy; 6 General Direction, ASL3 Genovese, Genoa, Italy

**Keywords:** diffuse pain syndrome, fatigue, quality of life, widespread pain

## Abstract

**Objectives:**

Fibromyalgia symptoms have a significant impact on the quality of life and respond poorly to medications. It has been hypothesized that the use of low-energy pulsed electromagnetic field (PEMF) induces neuroprotective effects that may interfere with pain perception. We explored the efficacy of PEMF in patients affected by fibromyalgia.

**Methods:**

Twenty-one females (median age 59 years, interquartile range [IQR] 16.5) affected by fibromyalgia were randomized to receive pulsed electromagnetic field-triple energy pain treatment (PEMF-TEPT) or placebo at T0 and at 4 weeks and 8 weeks. Fibromyalgia impact questionnaire (FIQ), widespread pain index (WPI), visual analog score (VAS) pain, symptom severity (SS) scale, and short form 36 (SF-36) health survey questionnaire have been evaluated.

**Results:**

Patients in the PEMF-TEPT group had a significantly higher reduction of WPI compared to placebo (mean difference −12.90 ± standard deviation [SD] 5.32 vs. −1.91 ± 4.55, difference in difference [DD] of −10.99; *P* < 0.001), of SS score (−4.10 ± 4.85 vs. −2.00 ± 2.32; DD = −2.1; *P* < 0.05), of VAS pain (−48 ± 30.75 vs. −16.82 ± 23.69; DD = −31.18; *P* < 0.01). They also reported a higher improvement of FIQ and SF-36, albeit not reaching statistical significance.

**Conclusion:**

In our pilot controlled study, PEMF-TEPT appeared to be safe and improved fibromyalgia symptoms.

## Background

Fibromyalgia affects between 0.4% and 8.8% of the general population, with a marked female predominance, and is characterized by chronic widespread pain associated with dysesthesia, paresthesia or sensation of burning, tingling or numbness, and stiffness, as well as fatigue, poor sleep quality, cognitive impairments in memory and concentration, headache, mood disorders, and bowel alterations.^[1]^ The diagnosis of fibromyalgia is predominantly clinical and does not exclude the presence of other causes for pain.^[2]^ While the etiology and pathogenesis of fibromyalgia remain unknown, some lines of evidence point to a role for the central nervous system in pain amplification and in the development of other symptoms. At the time of onset, some patients start complaining of regional pain and later develop widespread pain, even in the absence of an identifiable input, with a top-down process, while others have a definite disease (i.e., osteoarthritis) and pain becomes generalized afterward, with a bottom-up pattern, possibly related to altered nociception and nerve connectivity.

From a therapeutic standpoint, fibromyalgia management is aimed at improving quality of life but anti-inflammatory and analgesic drugs such as opioids have marginal effects and are generally not tolerated.^[3]^ Despite the use of muscle relaxants and antidepressants along with non-pharmacological treatments,^[4]^ symptoms are often uncontrolled, not allowing a good quality of life.^[5]^ Among alternative treatments, transcranial magnetic stimulation^[6]^ and transcutaneous electrical nerve stimulation^[7]^ have been proposed based on data coming from heterogeneous studies with high risks of bias.^[8, 9]^

Low-energy pulsed electromagnetic field (PEMF) is based on the use of magnetotherapy, which produces modulatory and neuroprotective effects^[10]^ while promoting osteogenesis and angiogenesis.^[11]^ While effective in preventing falls in fragile individuals,^[12]^ PEMF had moderate beneficial effects in treating pain from osteoarthritis when applied to the whole body or to a specific joint^[13]^ or rheumatoid arthritis and fibromyalgia.^[14, 15]^ In a randomized, double-blind, sham-controlled trial, PEMF administered to the whole body was effective in improving function, pain, fatigue, and global status in patients with fibromyalgia, and the results were maintained at 12 weeks,^[16]^ but in another randomized controlled trial, the PEMF system called Bio-Electro-Magnetic-Energy Regulation (BEMER) had no effect on fibromyalgia.^[17]^ As previous studies applied PEMF to the brain or to the whole body with conflicting results, and based on the challenges posed by the evaluation of patient-reported outcomes in fibromyalgia, we performed a randomized single-blind controlled pilot study to determine the potential benefits of PEMF on different disease symptoms.

### Patients and Methods

#### Subjects

Twenty-one women affected by fibromyalgia based on the 2010 American College of Rheumatology (ACR) classification criteria^[15]^ were enrolled if they fulfilled the following inclusion criteria: a diagnosis of fibromyalgia for at least 1 year, visual analog scale (VAS) score for pain ≥4 in the 2 weeks before the enrollment, and absence of any chronic analgesic treatment (Table 1). Pregnant or breast-feeding patients were excluded, along with patients with a coexisting inflammatory musculoskeletal condition. All patients were asked not to take pain medications (including non-steroidal anti-inflammatory drugs (NSAIDs), tramadol, opioids, pregabalin, and gabapentin) or antidepressants during the 7 d prior to enrollment and throughout the 8 weeks of the study to avoid interference with clinical outcomes, while the use of acetaminophen was allowed. The study was carried out in accordance with the declaration of the World Medical Association, procedures were in accordance with the Helsinki Declaration, and the ASL3 Genova ethics committee approved the protocol, with all patients signing an informed consent before enrollment.

**Table 1:** Inclusion and exclusion criteria for women with fibromyalgia included in the present study.

**Inclusion criteria**
Fibromyalgia diagnosis for at least one year, according to the 2010 ACR criteria
VAS pain score≥4 in the two previous weeks
Age between 18–65 years
Skin integrity in the application area of electrodes
No pregnancy and breastfeeding
Cognitive integrity
Formal consent to study participation
**Exclusion criteria**
A current diagnosis of infections or musculoskeletal inflammatory conditions
Use of NSAIDs, opioids, anti-depressants, beta-blockers in the 7 days before enrollment
Chronic or acute pulmonary, hematologic or kidney diseases
Active malignancy
Life expectancy < 6 months
Participation to other experimental studies in the month prior to the enrollment
Severe heart conditions or pacemaker
Chronic abuse of illicit drug and alcohol
Neuropsychiatric disorders

#### Study Protocol

**Figure 1:**
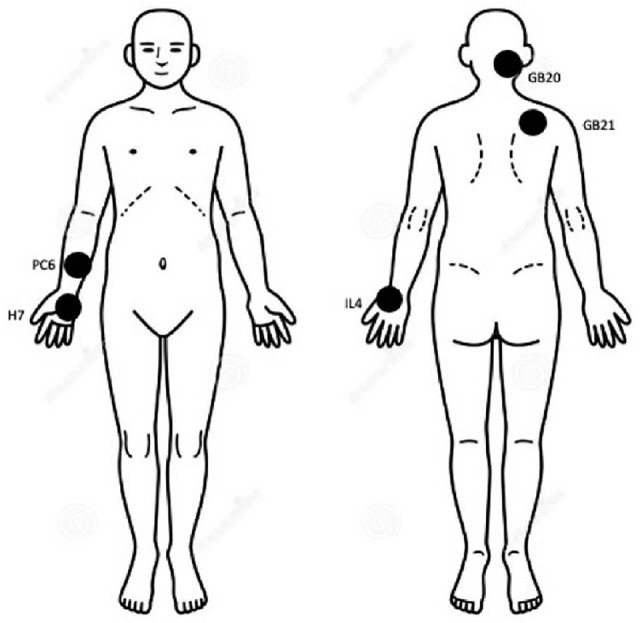
Selected target points for PEMF application in the present study, as described in the available literature.^[34]^ Briefly, points are located as follows: LI 4 (He Gu) at the height of the midpoint of the second metacarpal joint, at the peak formed by the first dorsal interosseous muscle where the thumb approaches the index finger; GB20 (Feng Chi) below the occipital bone, in the depression between the trapezius and sternocleidomastoid muscles; GB21 (JianJing) at the highest point of the shoulder halfway between the acromion and the spinous process of C7; HT7 (Shen Men) on the flexion fold of the wrist, between the pisiform bone and the ulna, in the depression medial to the tendon of the ulnar carpal flexor muscle; PC6 (Nei Guan) 2 cm above the wrist flexion fold, between the tendons of the radial flexor carpus and palmar long muscles. PEMF, pulsed electromagnetic field.

We performed a single-center randomized single-blind controlled pilot trial to determine the effect of low-energy PEMF therapy applied to target points on fibromyalgia symptoms (Figure 1).

The chosen points for PEMF application were based on the acupuncture experience in fibromyalgia^[18, 19]^ and the observed response of the parasympathetic and sympathetic system. ^[20, 21]^ Moreover, they were chosen because they are easy to identify and PEMF stimulation can be quickly applied.

Patients with fibromyalgia were consecutively enrolled between May and August 2019 and randomly allocated 1:1 to receive either pulsed electromagnetic field-triple energy pain treatment (PEMF-TEPT) (*n* = 10, herein referred to as active treatment) on pre-specified points or minimal intensity applied on scrambled points (*n* = 11 controls) for 20 min at baseline (W0) and after 4 weeks (W4) and 8 weeks (W8) (end of follow up: October 2019).

**Figure 2:**
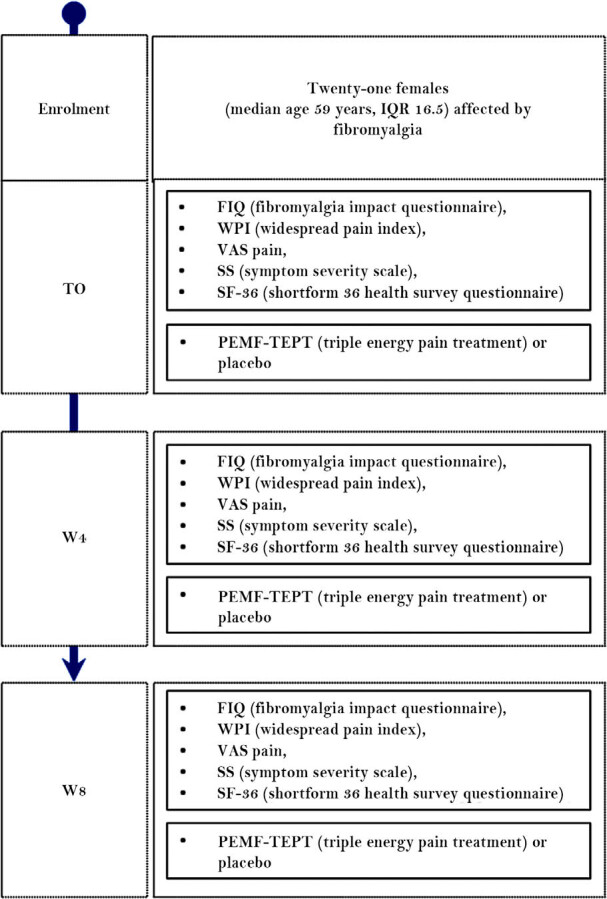
Study flow. For each timepoint treatment, scores assessed are specified. IQR, interquartile range; T, timepoint; VAS, visual analog scale; W, week.

Randomization for treatment or placebo was established by the device NewSunrise 280 P (THS – Therapeutic Solutions, Milan, Italy) and disclosed at analysis of data. Throughout the study, a consecutive number was assigned to each patient and also inserted in the device before starting a treatment session. This ensured that each patient received the same type of treatment in the different sessions and remained blind on the treatment allocation. In all cases, the intervention was provided by a trained rheumatologist in individual office sittings in a rheumatologic outpatient clinic in Genova, Italy, and attendance was verified each time. Outcome measures were recorded at each timepoint by the same rheumatologist. The primary outcome was the change observed in VAS pain, while secondary outcomes included other indexes with an impact on quality of life, such as fibromyalgia impact questionnaire (FIQ), widespread pain index (WPI), symptom severity (SS) scale, and short form 36 (SF-36) health survey questionnaires. Figure 2 illustrates the study flow. Adverse events were recorded at all timepoints using a clinical evaluation through history and physical examination.

#### Statistical Analysis

A difference in difference (DD) approach was used to evaluate the treatment effect on each response variable. In particular, for each patient, we calculated the difference between the value assumed by the response variable at time W8 and the same quantity at time T0. These variations were compared to the average between Placebo and Treated groups by performing 2 independent sample *t*-tests for difference in means (difference tested on differences). Formally, let *Y*_i,t,g_ be the value assumed by the response variable *Y* for a specific patient *i* who belongs to the treatment group *g* (Placebo and Treated), measured at time *t* (T0 and W8). We calculated the quantities Diff_i,g_ = *Y*_i,W8,g_ – *Y*_i,T0,g_ and compared them to the average between the 2 treatment groups (Placebo/Treated). All the assumptions of the *t*-test have been met and checked before performing it (i.e., normality has been checked via Shapiro–Wilk normality test and homoscedasticity via *F*-test on variance comparison).

## Results

The study included 21 female patients (mean ± SD age, 59 ± 17 years) diagnosed with fibromyalgia, and Table 2 illustrates the baseline features of patients randomized to the treatment groups, which did not differ significantly in terms of age or other clinical features.

**Table 2:** Clinical features of patients with fibromyalgia enrolled in the study, treated with PEMF or placebo. Continuous variables are expressed as mean ± standard deviation.

	**Total (*n*=21)**	**Active PEMF (*n*=10)**	**Controls (*n*=11)**	***P*-value**
Female sex (*N*/%)	21 (100%)	10 (100%)	11(100%)	–
Age (years, mean±SD)	59±17	55±9	62±10	0.10
Smoke (*N*/%)	6 (29%)	4 (40%)	2 (18%)	0.53
Osteoporosis (*N*/%)	4 (19%)	1 (10%)	3 (27%)	0.65
Osteoarthritis (*N*/%)	3 (14%)	2 (20%)	1 (9%)	0.92
Hypertension (*N*/%)	4 (19%)	2 (20%)	2 (18%)	0.65
Depression/anxiety (*N*/%)	5 (23%)	2 (20%)	2 (18%)	0.65
Insomnia (*N*/%)	3 (14%)	1 (20%)	2 (18%)	0.92
Autoimmune thyroid disease (*N*/%)	5 (23%)	1 (20%)	3 (27%)	0.65
Irritable bowel disease (*N*/%)	1 (4%)	0	1 (9%)	0.96
Gastroesophageal reflux disease (*N*/%)	3 (14%)	1 (20%)	3 (27%)	0.65
Allergies (*N*/%)	2 (9%)	0	2 (18%)	0.50
Referred use of psychoactive drugs (*N*/%)	11 (52%)	6 (60%)	5 (45%)	0.81
Referred use of NSAIDs^*^ (*N*/%)	5 (23%)	3 (30%)	2 (18%)	0.90
Referred use of pain medications^*^ (*N*/%)	12 (57%)	7 (70%)	5 (45%)	0.48

* *these treatments had to be withdrawn 7 days prior to enrollment.*

*P*-values indicate comparisons of clinical features between PEMF treated patients and control patients; *t*-test was used for continuous variable and Fisher's exact test for dichotomous variables.

**Figure 3:**
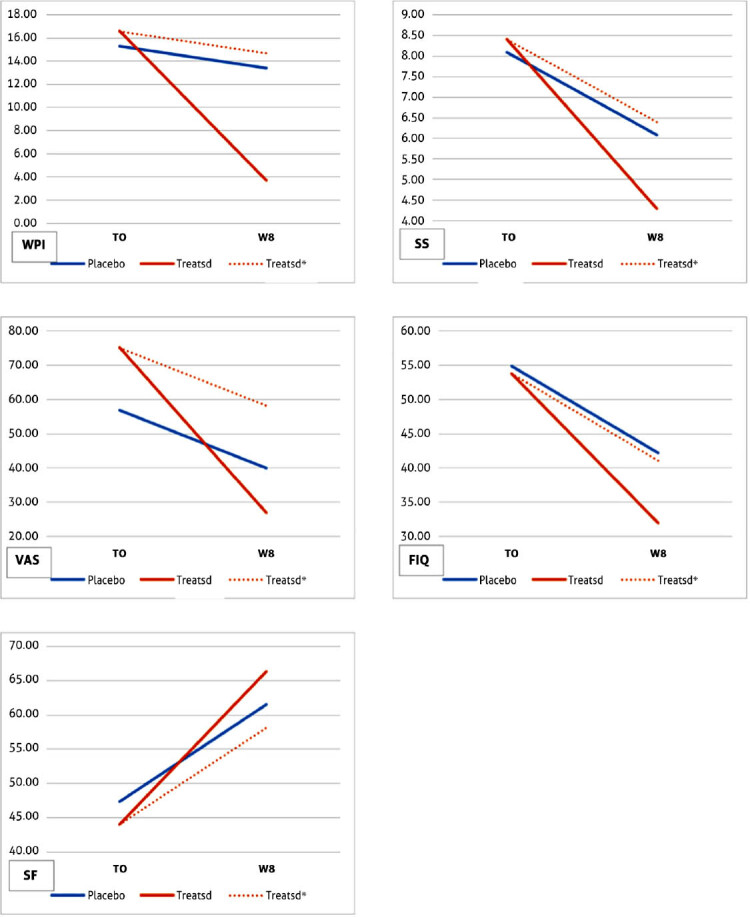
WPI, SS score, VAS for pain, SF-36 score, and FIQ score changes in patients receiving the active treatment and placebo (dotted lines represents the expected changes in the active treatment group if no effect was observed). FIQ, fibromyalgia impact questionnaire; SS, symptom severity; VAS, visual analog score; WPI, widespread pain index.

Table 3 summarizes the outcomes of DD analysis. For each response variable and for each group (Placebo and Treated), the average value at the beginning of the experiment (T0), the average value after 8 weeks (W8), the mean difference, and the standard deviation (SD) of the difference are reported. On the right panel of the table, the DD values used to evaluate the treatment effects are reported with the corresponding *P*-values of the one-sided *t*-test. Changes between baseline and W8 for WIP, SS, and VAS pain scores are illustrated in Figure 3. Between baseline and W8, patients receiving the active treatment with PEMF-TEPT on the target points (*n* = 10) illustrated in Figure 1 had a significantly deeper reduction of WPI (mean ± SD −12.90 ± 5.32 vs. −1.91 ± 4.55 in controls with an observed and significant DD = −10.99; *P* < 0.001) and significantly more pronounced reduction in the SS (−4.10 ± 4.85 vs. −2.00 ± 2.32 in controls; DD = −2.10; *P* < 0.05) and VAS (−48 ± 30.75 vs. −16.82 ± 23.69 in controls, DD = −31.18; *P* < 0.01) scores.

**Table 3:** Difference in difference analysis of the clinical outcomes.

**Response variable**	**Placebo group**			**Treated group**			**DD: Treated - Placebo**		
		
	**T0**	**W8**	W8 - T0^*^	**T0**	**W8**	W8 - T0^*^	**mean**	**SE**	***P*-value**
WPI	15.27	13.36	−1.91 (4.55)	16.60	3.70	−12.90 (5.32)	−10.99	2.15	< 0.01
SS score	8.09	6.09	−2.00 (2.32)	8.40	4.30	−4.10 (2.85)	−2.10	1.13	0.0392
VAS pain	56.82	40.00	−16.82 (23.69)	75.00	27.00	−48.00 (30.75)	−31.18	11.91	0.0085
FIQ	54.88	42.20	−12.67 (19.06)	53.74	31.96	−21.78 (19.29)	−9.11	8.38	0.1452
SF-36	47.39	61.49	14.10 (13.64)	44.07	66.39	22.32 (16.85)	8.22	6.66	0.1162

* *Mean difference; SD of the difference in brackets.*

WPI: widespread pain index; SS score: symptom severity scale; VAS: visual analog scale; FIQ: fibromyalgia impact questionnaire; SF-36: short form 36 health survey questionnaire.

Patients receiving active treatment also had improvements between baseline and W8 in FIQ and SF-36 scores, albeit not reaching statistical significance (Table 3).

Both active and placebo treatments were very well-tolerated and no side effects were noted in either group; all 21 patients completed the 8-week period of the study.

## Discussion

Chronic widespread pain has become one of the major reasons for physician consultation in the general population,^[15]^ and fibromyalgia is a predominant cause with an enormous impact on disability and quality of life. Despite numerous studies and recent recommendations,^[15]^ the current medical management of fibromyalgia is largely unsatisfactory and there is a need for new therapeutic options. Data from our pilot study with a randomized single-blind controlled design on a limited number of subjects suggest that the use of PEMF could provide benefits over an 8-week period on the major indices of fibromyalgia activity.

PEMF is based on the use of magnetic fields that can be applied to the brain, to the whole body, or to specific sites, having a more general effect on pain. ^[13,14,15]^ No relevant side effect has been described so far and the treatment is usually well-tolerated.^[22]^ We observed a significant effect of PEMF on WPI, SS, and VAS scores in treated patients compared to placebo. These observations support previous reports of possible beneficial effects of electromagnetic fields on human pain.^[15]^

The mechanisms by which PEMF may be beneficial in fibromyalgia can only be speculated, and were not explored in the current study. We should observe, however, that this lack of background rationale is frequently encountered in most exploratory studies in fibromyalgia as the understanding of the disease pathogenesis remains elusive and its management challenging.^[23]^ One major hypothesis is that PEMF changes brain waves^[24]^ and reduces hypoxic damage in neuron-like and microglia cells.^[25]^ PEMF induces a direct cellular response, promoting proliferation and differentiation of the human osteoblast, by increasing mitochondrial activity and activating the extracellular signal-regulated kinase (ERK) 1/2 signaling cascade.^[26]^ Furthermore, PEMF can promote anti-oxidative defense mechanisms and mitochondrial repair in osteoblasts.^[27]^ It is of note that these mechanisms have been implicated in the pathogenesis of fibromyalgia, including brain wave alterations at electroencephalography,^[28]^ mitochondrial damage associated with reduced nitric oxide levels in the blood, causing an impaired circulation which is involved in fatigue, a major fibromyalgia symptom,^[29]^ and oxidative stress.^[30]^

We observed that the application of PEMF-TEPT on selected points can be helpful in the management of pain in fibromyalgia but also may have an impact on other less defined domains such as fatigue, anxiety, depression, and muscular stiffness, which impact the quality of life and also influence the patient's personal life in terms of their ability to work and be active socially. The changes in SS score, FIQ, and WPI are of particular importance in the management of fibromyalgia as these account for the predominant symptoms of fibromyalgia, i.e., pain, sleep disturbances, and cognitive impairment. As PEMF has been found to be helpful in the treatment of anxiety and depression in human studies,^[31]^ we are particularly intrigued by the effects of PEMF-TEPT on the scores resulting from the FIQ and the SF-36 questionnaires. These changes did not reach statistical significance in comparison with the control treatments, possibly due to the small sample size, or likely due to the fact that emotional features are included,^[32]^ which are marginally affected by PEMF.

Some issues need to be discussed to represent the strengths and weaknesses of our data well. First and foremost, the randomized single-blind design is of importance in fibromyalgia since the placebo and nocebo effects are relevant in these conditions,^[33]^ as illustrated by the amelioration observed in all readouts with the control treatment. We should note that the expected changes with the active treatment group, if no real effect due to PEMF was observed (represented by the dotted lines in Figure 3), differ from those actually observed, thus suggesting that the beneficial role of PEMF exceeds the placebo effect. Second, the small number of enrolled patients due to the inclusion of only women to minimize the variability of the cohort does not allow us to draw definitive conclusions, and a larger prospective study is needed to confirm these observations. The apparent limitation of the single-blind design was unavoidable since the treating physician had to be aware of the areas to which PEMF had to be applied, but the self-reported measures of efficacy limit the impact of this potential weakness.

Further important information could be obtained by comparing the effects of PEMF when applied on peripheral sites, on the brain, or on both, by investigating which method promotes better results.

In conclusion, we investigated the role of PEMF on specific body areas in fibromyalgia patients, supporting a safe therapeutic strategy which had already been tested in other mechanisms of chronic pain (e.g., osteoarthritis). The treatment appears to improve pain and fatigue and the general overall health status in patients affected by this chronic condition, in the absence of relevant side effects. PEMF can be considered a therapeutic choice in the management of fibromyalgia, and further studies are needed to assess whether specific categories of fibromyalgia patients could benefit more from this therapy.
